# Intrauterine Treatment of a Fetus with Familial Hypertrophic Cardiomyopathy Secondary to MYH7 Mutation

**DOI:** 10.1007/s00246-015-1250-1

**Published:** 2015-09-04

**Authors:** Meghan G. Hill, Mehtab K. Sekhon, Kathryn L. Reed, Caroline F. Anderson, Nydia D. Borjon, Jil C. Tardiff, Brent J. Barber

**Affiliations:** Department of Obstetrics and Gynecology, The University of Arizona, College of Medicine, 1501 N. Campbell Ave, 8th Floor, Tucson, AZ 85724 USA

**Keywords:** Fetal hypertrophic cardiomyopathy, Beta myosin mutation, Propranolol, Fetal echocardiography

## Abstract

There is no clear consensus on optimal management of fetuses affected by familial hypertrophic cardiomyopathy (HCM). Intrauterine treatment of the condition has not been attempted in any standardized fashion. We report the case of a fetus treated by maternal propranolol during the third trimester after septal hypertrophy and diastolic dysfunction was diagnosed on fetal echocardiogram. The pregnancy went successfully to term, and fetal septal hypertrophy was noted to improve prior to delivery.

## Case Report

Fetal hypertrophic cardiomyopathy (HCM) is most commonly seen in the setting of maternal diabetes mellitus [10, 11] and typically resolves spontaneously in the first few weeks after delivery. Rarely, fetal HCM may be secondary to a familial genetic mutation, classically a mutation in one of the contractile proteins of the cardiac sarcomere. In this situation, the condition can have serious long-term clinical consequences [5].

We report the intrauterine course and management of a baby girl born to a 30-year-old, G6P2 mother who initially presented for prenatal care at 7 weeks of gestation. There was no maternal history of diabetes mellitus, and gestational diabetes screening performed in the pregnancy was normal.

### Family History

The baby’s father and two older brothers carry a familial β-myosin heavy chain mutation—MYH7 Lys657Gln (K657Q)—resulting in HCM. The father was diagnosed with HCM as a child and underwent heart transplantation at age 33. The two brothers were diagnosed with HCM as neonates following normal fetal echocardiograms without evidence of fetal HCM. Their initial septal *Z*-scores at 1 day of age were +3.7 (oldest) and +3.0 (second brother). They both were started on propranolol as neonates and have been maintained on 3 mg/kg/day divided into three daily doses since that time. In both boys, a decrease was noted in the septal thickening with normalization of septal *Z*-scores by 9 months.

### In Utero/Fetal History

**Fig. 1:**
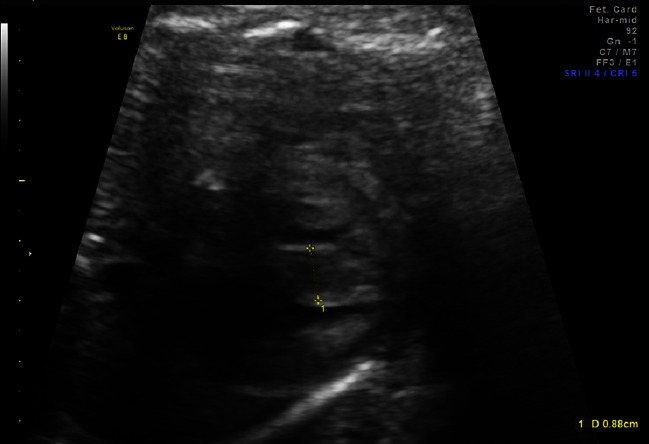
Fetal echo at 35-weeks estimated gestational age—interventricular septum in diastole—measuring 8.8 mm (*Z*-score +11.3)

**Fig. 2:**
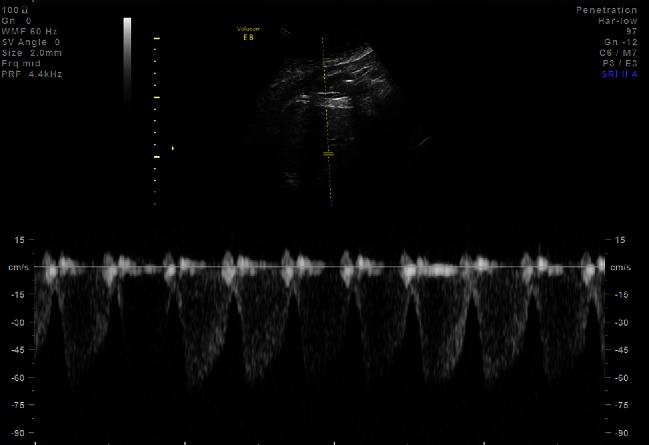
Ductus venosus waveform at 35-week estimated gestational age demonstrating an abnormal waveform with a decreased velocity in diastole. These findings are consistent with fetal diastolic dysfunction.

At 30 weeks of gestation, fetal HCM was suspected. At that time, fetal echocardiogram revealed mild thickening of the interventricular septum in diastole (4.7 mm; normal 2.5th–97.5th % = 1.8–3.6 mm) [3]. Repeated fetal echocardiography over the next several weeks revealed progressive septal thickening with a maximal septal measurement at 35-weeks of gestation of 8.8 mm (*Z*-score + 11.3; normal = 2.3–4.2 mm) [3] (Fig. 1). An abnormal ductus venosus waveform was also noted at 35 weeks of gestation with decreased late diastolic velocity during the a-wave of 15cm/sec (normal range at 35 weeks approximately 30-60cm/sec) [4]. (Fig. 2). We elected to initiate intrauterine treatment based on two factors: (1) fetal diastolic dysfunction and (2) the family history of improvement in septal thickening (potentially associated with beta-blocker treatment) noted in the two previous children with HCM. Therefore, maternal administration of propranolol was initiated (10 mg orally three times daily). Over the next several weeks, fetal echocardiograms were repeated and revealed decreased septal thickness: 7.8 mm at 37 weeks of gestation and 7.6 mm at 38 weeks. The ductus venosus waveform did not significantly change however and remained abnormal.

### Birth/Neonatal History

**Fig. 3:**
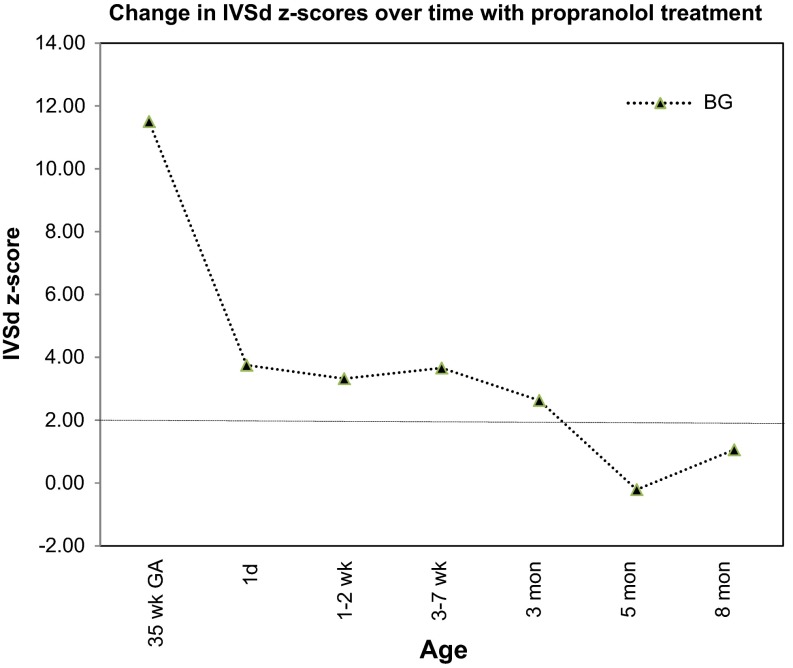
This figure shows the change in IVSd *Z*-scores of the index case over time with propranolol treatment. The IVSd *Z*-score for the index case at 35-week gestational age (GA) is also shown

The baby girl was delivered via repeat cesarean section at 39 weeks and weighed 3470 g, length 49 cm, and had Apgar scores of 9 and 9. The initial echocardiogram on the first day of life noted a diastolic septal measurement of 7.9 mm (*Z*-score +3.35). A small muscular VSD was also noted. She did well, was initiated on propranolol and discharged home on day three of life. She has been continued on propranolol (3 mg/kg/day in three divided daily doses) and, like her brothers, has had regression of septal hypertrophy and now has normal septal dimensions (Table 1; Fig. 3). Genetic testing confirmed that she shares the same β-myosin heavy chain mutation—MYH7 Lys657Gln (K657Q)—as her father and brothers.

**Table 1:** Change in intraventricular septal thickness in diastole (IVSd) with propranolol treatment for the index case. The IVSd *Z*-score was calculated with the Detroit database at www.parameterz.com. Fetal *Z*-scores were calculated from 2.5th to 97.5th percentiles in [3]

	35 weeks GA		1 day		1–2 weeks		3–7 weeks		3 months		5 months		7–8 months	
	Raw value	*Z*-score	Raw value	*Z*-score	Raw value	*Z*-score	Raw value	*Z*-score	Raw value	*Z*-score	Raw value	*Z*-score	Raw value	*Z*-score
BG														
IVSd (mm)	8.8	11.5	7.9	3.75	7.5	3.32	8.2	3.66	7.0	2.63	4.0	-0.21	5.4	1.06

## Discussion

Hypertrophic cardiomyopathy is the most common familial heart disease occurring in approximately 1/500 young adults [6]. Hypertrophic cardiomyopathy is inherited in an autosomal dominant fashion and carries significant clinical consequences including a risk of sudden cardiac death. The prevalence and clinical significance of fetal HCM is unknown. In HCM, ventricular systolic function is typically hyperdynamic, but ventricular relaxation is impaired. Increased ventricular diastolic filling pressure can decrease umbilical vein and ductus venosus flow into the fetal heart and is associated with elevated fetal central venous pressure, which may result in effusions, ascites and fetal demise [9]. Fetal diastolic dysfunction has been associated with poor outcomes including fetal hydrops [5, 9]. We observed a case of fetal familial HCM with diastolic dysfunction evidenced by an abnormal ductus venosus waveform. Secondary to the risk of hydrops and the previous family history of a positive response to beta-blockers, we elected to treat with maternal propranolol.

Beta-blocker treatment is recommended in the treatment of children and adults with symptomatic HCM [2], but no recommendations or previous reports of treatment of fetal HCM have been published. Beta-blockers reduce heart rate, decrease myocardial consumption and have a negative inotropic effect. Beta-blockade has been shown to improve echocardiographic parameters of LV diastolic function [1]. Treatment with beta-blockers in asymptomatic patients with HCM is not established; however, we elected to treat secondary to the observed abnormal parameters of fetal diastolic function and the risk of fetal hydrops/demise.

While specific response to beta-blocker treatment is not currently a documented feature of MYH7 HCM, beta-blocker treatment in childhood HCM has been associated with a reduction in cardiac hypertrophy and improved survival [8]. It has been suggested that the rapid growth of the heart in childhood may provide a favorable window of opportunity for medical treatment in HCM [8]. This may also apply to the rapidly growing fetal heart.

 The use of beta-blockers is not without concern in pregnancy. Beta-blockers are an FDA category C drug because of reported risks of in utero exposure, resulting in fetal growth restriction, heart defects, fetal and neonatal bradycardia, neonatal hypoglycemia and neonatal respiratory depression. Fortunately, we did not observe any of these fetal or neonatal complications.

While it is interesting to speculate on the encouraging early results noted with beta-blocker treatment in this family with an MYH7 mutation, it has been noted that HCM genotype–phenotype prognostic correlations are difficult. This is attributed to HCM being an “exceedingly complex biologic entity with vast genetic heterogeneity” [7].

Clinical trials of fetal HCM treatment would be difficult to accomplish, but we are encouraged by the results noted in this case and would recommend consideration of intrauterine beta-blocker treatment for a fetus with suspected familial HCM and evidence of diastolic dysfunction.
